# Comparison of Mutation Patterns in Full-Genome A/H3N2 Influenza Sequences Obtained Directly from Clinical Samples and the Same Samples after a Single MDCK Passage

**DOI:** 10.1371/journal.pone.0079252

**Published:** 2013-11-01

**Authors:** Hong Kai Lee, Julian Wei-Tze Tang, Debra Han-Lin Kong, Tze Ping Loh, Donald Kok-Leong Chiang, Tommy Tsan-Yuk Lam, Evelyn Siew-Chuan Koay

**Affiliations:** 1 Department of Pathology, Yong Loo Lin School of Medicine, National University of Singapore, Singapore, Singapore; 2 Department of Laboratory Medicine, National University Hospital, National University Health System, Singapore, Singapore; 3 Alberta Provincial Laboratory for Public Health, University of Alberta Hospital, Edmonton, Canada; 4 Department of Medical Microbiology and Immunology, University of Alberta, Edmonton, Canada; 5 Department of Zoology, University of Oxford, Oxford, United Kingdom; University of Edinburgh, United Kingdom

## Abstract

Human influenza viruses can be isolated efficiently from clinical samples using Madin-Darby canine kidney (MDCK) cells. However, this process is known to induce mutations in the virus as it adapts to this non-human cell-line. We performed a systematic study to record the pattern of MDCK-induced mutations observed across the whole influenza A/H3N2 genome. Seventy-seven clinical samples collected from 2009-2011 were included in the study. Two full influenza genomes were obtained for each sample: one from virus obtained directly from the clinical sample and one from the matching isolate cultured in MDCK cells. Comparison of the full-genome sequences obtained from each of these sources showed that 42% of the 77 isolates had acquired at least one MDCK-induced mutation. The presence or absence of these mutations was independent of viral load or sample origin (in-patients versus out-patients). Notably, all the five hemagglutinin missense mutations were observed at the hemaggutinin 1 domain only, particularly within or proximal to the receptor binding sites and antigenic site of the virus. Furthermore, 23% of the 77 isolates had undergone a MDCK-induced missense mutation, D151G/N, in the neuraminidase segment. This mutation has been found to be associated with reduced drug sensitivity towards the neuraminidase inhibitors and increased viral receptor binding efficiency to host cells. In contrast, none of the neuraminidase sequences obtained directly from the clinical samples contained the D151G/N mutation, suggesting that this mutation may be an indicator of MDCK culture-induced changes. These D151 mutations can confound the interpretation of the hemagglutination inhibition assay and neuraminidase inhibitor resistance results when these are based on MDCK isolates. Such isolates are currently in routine use in the WHO influenza vaccine and drug-resistance surveillance programs. Potential data interpretation miscalls can therefore be avoided by careful exclusion of such D151 mutants after further sequence analysis.

## Introduction

Influenza viruses obtained from infected human host specimens can be isolated using several different cell-lines. They include embryonated chicken eggs, *in vitro* monolayers of primary cell-line: rhesus monkey kidney (RhMK), and established continuous cell-lines: the African green monkey kidney (AGMK/Vero), Madin-Darby canine kidney (MDCK), mink lung epithelial (Mv1Lu), rhesus monkey kidney (LLC MK2), and buffalo green monkey kidney (BGMK) cell-lines [1]. Among these, the MDCK cells have been used extensively in various clinical diagnostic [1] and research [2-5] investigations of influenza viruses. It is particularly useful for the amplification of influenza viruses found in clinical samples [6] to produce sufficient amounts of virus for experimental research and distribution to other research laboratories [7-10].

Host-induced mutations induced during viral passaging have been reported sporadically [5,8,11-15]. Yet, despite the extensive use of MDCK cells in influenza research, there are no systematic studies of possible MDCK-induced mutations across the whole influenza genome. Only a few reports of MDCK-induced mutations in individual gene segments have been published [12,14,16]. These MDCK-induced mutations may have direct and significant impact on the data interpretation in studies related to viral molecular epidemiology [11], antigenicity and pathogenicity [7], and patterns of drug resistance [8-10,12,14]. For these studies, only the hemagglutinin (HA), neuraminidase (NA), and matrix protein (MP) genes were routinely sequenced [5,11,17]. An accurate characterization of the pattern of MDCK-induced mutations across the whole genome would improve the quality and accuracy of data interpretation of influenza virus mutation studies.

In this study, we performed an extensive genome sequence comparison between influenza A/H3N2 viral sequences obtained: 1) directly from, and 2) after isolation in MDCK cells, from each of the clinical respiratory samples. 

## Results

### Viral culturing and sequencing

A total of 77 influenza A/H3N2 clinical samples with cycle threshold (Ct) values of 15.34-33.22 (mean: 23.91; SD: 3.89) were included in this analysis. For each of these samples, two full influenza genomes were obtained: one from virus obtained directly from the clinical sample and one from virus that was cultured once in the MDCK cell-line. These relatively high viral load samples (Ct < 33.22; > 408 viral copies/μL of RNA extract) were used to permit full genome sequences to be obtained from both of these virus sources.

In addition, to test the reproducibility of the pattern of MDCK-cultured viral sequences, 20 replicates of a clinical sample with a high viral load (7x10^6^ viral copies/μL of RNA extract) of influenza A/Singapore/H2011.704/2011(H3N2) was cultured simultaneously. All cultured samples analyzed in this study had only single passage history. 

The complete genome sequences of all the paired clinical and cultured influenza A/H3N2 (n= 77 direct from source + 77 MDCK-cultured = 154) were generated and subjected to an exhaustive phylogenetic analysis to screen for any artifacts and/or sequence mosaics that may have been induced by the sequencing and other laboratory methods or contaminants [18]. In brief, this was performed by running all of these sequences through a suite of phylogenetic programs designed to detect the presence of any recombination breakpoints in these sequences, as described by Lam et al. (2013).

After completing these analyses, all of the sequences were submitted to the NCBI GenBank. Twenty-one of the 154 genome sequences (7 from clinical samples and 14 from isolates, from different strains) had been submitted earlier from a previous study [19]; the remaining 133 genome sequences, with accession codes of KF014126-KF015189, were submitted in June 2013.

To evaluate the consistency of the Sanger method, the extracted RNA from a clinical sample as well as a cultured isolate with high viral loads were repeatedly sequenced - ten replicates each. The results of these twenty replicate experiments did not show any discrepancy in the sequence call rate for each set of replicates.

### Sequence comparison between clinical and MDCK-cultured samples

Comparison of the full genome sequences between the clinical sample and its corresponding isolate showed that 32 out of 77 (42%) isolates had undergone MDCK-induced mutations. The affected nucleotide and amino acid (aa) positions of each of the affected isolates (including synonymous and missense changes) are summarized in the Table 1. 

**Table 1:** Summary of MDCK-mediated mutations for each of the affected influenza A/H3N2 virus isolates examined in this study.

Strain ID	Nucleotide change^a^	Amino acid change
**Segment 1/ polymerase basic 2 (PB2)**		
C2011.422	2118A>R	I697M
C2011.496	600A>M	E191D
C2011.647	1287C>T	F420^c^
	1362T>C	L445^c^
H2009.518	1724A>T	Q566L
H2010.619	1589Y>C	I/T521T
H2010.822	2200C>Y	L725^c^
H2011.507	2013T>W	T662^c^
H2011.797	1836G>K	M603I
**Segment 4/ hemagglutinin (HA)**		
C2009.485a	572C>M	N165N/K^b^
C2011.301	739C>Y	P221P/L^b^
C2011.564	489G>K	A138A/S^b^
C2011.614	739C>Y	P221P/L^b^
H2009.679	729G>R	G218G/R^b^
H2010.559C	739C>Y	P221P/L^b^
	744G>R	V223V/I^b^
H2011.797	729G>R	G218G/R^b^
H2011.808bC	644G>R	K189^b^,^c^
**Segment 5/ nucleoprotein (NP)**		
C2011.471	123G>R	R26^c^
H2010.822	1262C>Y	T406T/I
H2011.570	1002C>Y	N319^c^
**Segment 6/ neuraminidase (NA)**		
C2009.863	268A>R	E83^c^
	470G>R	D151D/N
C2010.937V	121A>R	T34^c^
	471A>R	D151D/G
C2011.301	470G>R	D151D/N
C2011.362V	470G>R	D151D/N
C2011.452	470G>R	D151D/N
C2011.471	470G>R	D151D/N
C2011.477	470G>R	D151D/N
C2011.493	471A>R	D151D/G
C2011.496	470G>R	D151D/N
C2011.507V	470G>R	D151D/N
C2011.573	471A>R	D151D/G
C2011.614	471A>R	D151D/G
C2011.641	471A>R	D151D/G
C2011.825	470G>R	D151D/N
H2010.321C	470G>A	D151N
H2010.559C	251T>Y	C78C/R
H2010.797	471A>R	D151D/G
H2011.463	1066G>R	V349^c^
H2011.482	470G>R	D151D/N
H2011.507	395C>Y	P126P/S
H2011.570	471A>R	D151D/G
**Segment 8/ non-structural (NS)**		
C2011.362V	592R>G	NS1: D/G189G; NS2: I/V32V
C2011.573	578R>A	NS2: G/D27D
H2010.559C	335C>Y	NS1: F103^c^

Alternatively spliced mRNAs of the non-structural protein gene allows translation of two alternate proteins, non-structural protein 1 (NS1) and non-structural/ nuclear export protein (NS2/NEP). The non-ATCG letters coded for ambiguous/degenerate nucleotide sequences, i.e. “R” - “A and G”, “M” – “A and C”, “Y” – “C and T”, “W” – “A and T”, and “K” – “G and T”.

^a^Nucleotide numbering based on influenza A/Singapore/C2009.458V/2009(H3N2), GenBank accession: KF015129, KF014198, KF014597, KF014464, and KF014730.

^b^Amino acid numbering without including signaling peptide (i.e. the first 16 amino acids)

^c^Change of nucleotide or amino acid that resulted in synonymous mutation.

Other than the recurrent mutations found in the HA and NA segments, all individual missense mutations occurred just once. The emergences of A138S, N165K, G218R, P221L, and V223I were observed within the HA segment, particularly at the HA_1_ domain (residues 1-328), with frequencies of 1, 1, 2, 3, and 1, respectively. Out of the 77 isolates, amino acid changes to N or G at position D151 were observed in the NA segment with frequencies of 18 (23%), with the occurrence of D151N and D151G recorded at frequencies of 11 (14%) and 7 (9%). In contrast, none of the paired NA sequences obtained directly from the viruses in the clinical samples contained this D151 mutation. All the patients with D151 MDCK-induced mutations had no prior antiviral treatment history. 

The results from the single sample that was cultured in MDCK cells in 20 replicates, five (25%) had acquired one MDCK-induced mutation, respectively: one T148T/I and two D151D/N mutations in the NA segment, one R348K in the nucleoprotein (NP) segment, and a synonymous mutation at N312 of the polymerase basic 1 (PB1) segment.

### Association with Ct values

The mean of the Ct of samples that developed MDCK-induced mutations versus those that did not, were: 24.08 (range: 17.45-30.00) versus 23.79 (15.34-33.22), respectively. There was no statistically significant difference (*p* = 0.745; 2 Sample T-test) in the Ct values between the samples that contained and those that did not contain any MDCK-induced mutations. This suggests that the presence or absence of MDCK-induced mutations is not a viral load-dependent phenomenon in these clinical influenza A/H3N2 positive samples.

### Association with sample sources

Out of the 77 cultured samples sequenced, 44 were collected from hospitalized in-patients (n=37) or out-patients (n=7) seen at a tertiary-care academic medical center, the National University Hospital (NUH) of Singapore. The remaining were samples received from its affiliated primary-care clinics (n=33). No statistically significant difference (*p* = 0.0638; Fisher’s exact test) was found between the proportion of the samples containing MDCK-induced mutations collected from the in-patients (11/37, 30%) and from the outpatients, including those seen in the community primary-care clinics and the hospital out-patients (21/40, 53%). The 7 hospital out-patients were included with the community cohort because, in essence, the patients attending both types of out-patient clinics would have arrived in those clinics directly from the community population.

### Variation in residue 151 of NA in current influenza deposits

A total of 7229 complete NA sequences of influenza A/H3N2 collected from 2004-2012 were downloaded from the GISAID EpiFlu database (http://platform.gisaid.org/; last accessed 15 August 2013). The aa variations detected at residue D151 of these NA sequences are summarized in Table 2. Of the 7229 NA sequences, 2729 (38%) and 736 (10%) were obtained from MDCK-cultured isolates and primary clinical samples, respectively. 

**Table 2:** Total number of complete NA segments sequenced from different sample source types (n = 7306, including the 77 influenza A/H3N2 virus samples from this study collected between 2009 and 2011: 1^st^ row, In-house MDCK-cultured; and 7229 external samples from GISAID EpiFlu database collected between 2004 and 2012: 2^nd^-13^th^ rows, as summed up in the last row) and summary of amino acid variations detected at residue D151 of NA segments in respective sample source type (number and percentage of affected isolates).

Sample type^1^	Total deposits	D151D/N^2^	D151N^3^	D151D/G^2^	D151G^3^	D151D/E^2^	D151E^3^	D151D/V^2^	D151V^3^	D151D/A^2^	D151A^3^	D151N/T^2^	D151?^4^	Total & % (in brackets) of affected isolates
In-house MDCK-cultured	77	10 (13%)	1 (1%)	7 (9%)	0	0	0	0	0	0	0	0	0	18 (23.4%)
MDCK-cultured	2729	383 (14%)	51 (2%)	238 (9%)	50 (2%)	4 (0.1%)	20 (1%)	3 (0.1%)	2 (0.1%)	6 (0.2%)	2 (0.1%)	1	123	883 (32.4%)
Clinical sample	736	2 (0.3%)	0	0	0	0	0	0	0	0	0	0	0	2 (0.3%)
RhMK-cultured	122	0	0	0	0	0	0	0	0	0	0	0	0	0
Egg-cultured	249	2 (0.8%)	0	1 (0.4%)	0	0	0	0	0	0	0	0	0	3 (1.2%)
Spfck-cultured	12	0	0	0	0	0	2 (17%)	0	0	0	0	0	0	2 (16.7%)
MEK-cultured	29	0	0	0	0	0	0	0	0	0	0	0	0	0
CaCo-2-cultured	6	4 (67%)	0	1 (17%)	0	0	0	0	0	0	0	0	1 (17%)	6 (100%)
LLC-MK2-cultured	1	0	0	0	0	0	0	0	0	0	0	0	0	0
Vero cells-cultured	2	0	0	0	0	0	0	0	0	0	0	0	0	0
MDCK- and Egg- cultured	7	0	0	0	0	0	0	0	0	0	0	0	0	0
R-Mix- and RhMK- cultured	15	0	0	0	0	0	0	0	0	0	0	0	0	0
Non-specified	3321	532 (16%)	25 (0.8%)	318 (10%)	27 (0.8%)	7 (0.2%)	14 (0.4%)	22 (0.7%)	7 (0.2%)	3 (0.1%)	1	0	86 (3%)	1042 (31.4%)
Total excluding in-house MDCK-cultured samples	7229	923	76	558	77	11	36	25	9	9	3	1	210	1938 (26.8%)

^1^Abbreviation: MDCK, Madin-Darby canine kidney; RhMK, primary rhesus monkey kidney; SpfCk, specific-pathogen-free chick kidney; MEK, monkey epithelial kidney; Caco-2, Human colonic carcinoma cell; LLC-MK2, rhesus monkey kidney epithelial

^2^Number of D151 variants that exhibit both wildtype and mutant amino acids (mixes) at the same position of the NA sequence.

^3^Number of D151 variants that exhibit mutant amino acid only.

^4^Number of mutants that contain codons with ambiguous nucleotide bases which exhibit more than 2 possible amino acids.

Overall, the aa variations at position 151 on the NA sequence were more commonly found in the MDCK-cultured isolates. During the study period (2009-2011), the proportion of D151 mutations in the MDCK-cultured viruses derived from the EpiFlu database (327/1745, 19%) were similar to that detected in this study (18/77, 23%) (*p* = 0.300). Of the D151 mutations deposited in the EpiFlu database, 150 (9%) were D151N, 143 (8%) were D151G, and 34 (2%) were a mixture of both D151N and D151G.

**Figure 1:**
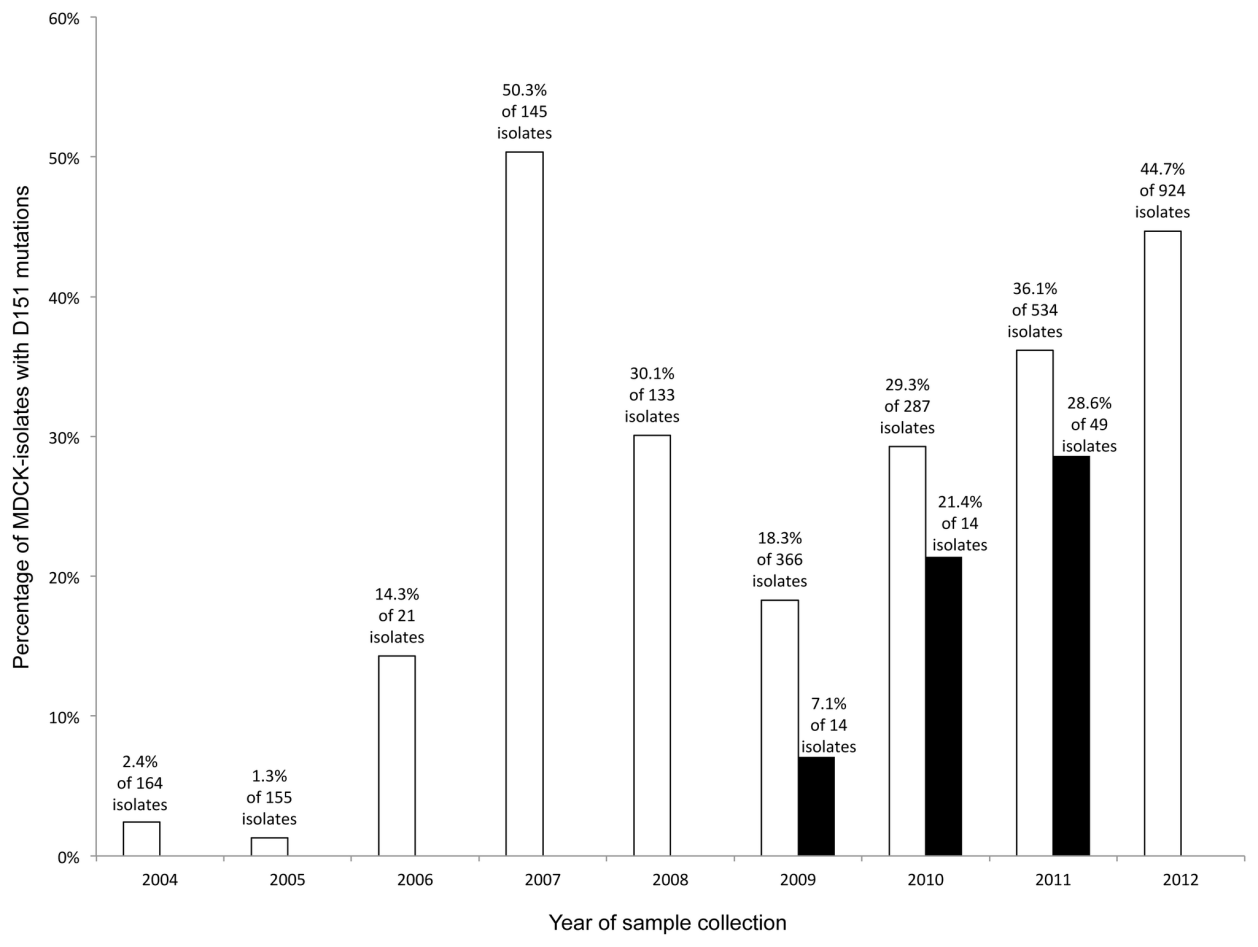
Distribution of D151 mutations isolated from MDCK-cultured influenza A/H3N2 viruses, according to the year of sample collection. The white and black columns represent the percentages of D151 mutations in MDCK-cultured isolates deposited in GISAID EpiFlu database (n=2729, 2004-2012) and from this study (n=77, 2009-2011), respectively. The relative trend in an increasing proportion of in-house MDCK-cultured isolates collected from 2009-2011 is similar to that for the GISAID sequences.

A total of 883 (32.4%) out of 2729 MDCK-cultured isolates collected during 2004-2012 had an aa change at position 151. By contrast, only 2 (0.3%) out of 736 clinical samples were found to carry such aa change. The distribution of the proportion of D151 mutation in the MDCK-cultured isolates over year of sample collection showed climaxes reached in 2007 and 2012 (Figure 1). A similar trend was observed from the in-house MDCK-cultured isolates collected from 2009-2011 in this study.

## Discussion

The results of this study demonstrate that in about 42% of clinical samples containing seasonal human influenza A/H3N2 virus, culturing in MDCK cells can lead to the emergence of variants carrying substitutions in most of the viral segments. To our knowledge, this is the first extensive report that has documented MDCK-induced mutation patterns at the whole genome level for human influenza A/H3N2 obtained from clinical diagnostic samples. The pairwise comparison of genomic sequences generated from MDCK-cultured samples and their matching primary clinical samples revealed a sporadic pattern of mutations in PB2, NP, and NS segments but not for the HA and NA segments (Table 1). 

### MDCK-induced changes in the HA protein

Interestingly, all five residue changes in HA protein were found within the HA_1_ domain only. In particular, four out of the five residue changes (A138S, G218R, P221L, and V223I) were within or proximal to the receptor-binding sites, particularly at the 130- and 220- loops [20,21]. The other (N165K) was located at the antigenic site of the virus [21]. 

The A138S mutation can emerge *in vivo* in immunocompromised patients and *in vitro* in MDCK cultures. Both have been found to cause total loss of receptor binding ability to the SAα2,3 Gal linkage [22-24]. Mutations at the HA_1_ 218 position, which is in the vicinity of the receptor-binding site, have also been reported to be associated with changes of receptor-binding specificity [25], as well as increased viral pathogenicity related to viremia leading to severe lung, spleen, intestine, brain, and heart infections, when tested in a mouse model [26]. In the similar study, the G218R mutation was found to reduce the SAα2,6 Gal linkage-binding affinity [25]. 

The V223I mutation was found to be induced during the passage of influenza viruses using embryonated chicken eggs [27]. This mutation has been shown to reduce the binding of HA, which seems to give it some resistance to the action of NA inhibitors (NAIs) by allowing the virus to be released from cells with less dependence on the NA enzyme [28]. However, this may not be the complete explanation as an immunocompromised child infected with a virus containing this mutation was found to exhibit susceptibility to zanamivir [29]. 

To date, no published data about P221L is available yet in the literature. However, the close proximity of this mutation to the receptor-binding sites may suggest similar alterations on receptor-binding specificity and/or affinity, as mentioned earlier. 

The N165 residue, which is located on the membrane-distal surface of HA, was identified as one of the oligosaccharide attachment/glycosylation sites that determine the viral antigenicity and assist in host-immune escape [30,31]. 

### MDCK-induced changes in the NA protein

From 2009-2011, emergences of G and N at residue D151 were detected in the NA segment with a frequency of 18 out of the 77 isolates (23%). Support for these findings is found in the 1745 EpiFlu MDCK-cultured sequences collected from the same period, where a similar proportion, 327/1745 (19%) demonstrated these same D151G/N mutations (*p* = 0.300). This suggests that the D151G/N mutation may be a marker of MDCK-cultured isolates, as noted previously [7,8], in approximately 20% of the samples, although in the majority of cultured isolates, this mutation is not present. 

It is interesting to note that the number of MDCK-induced mutations in these samples is similar to that induced in a single sample split into 20 aliquots and cultured in MDCK cells (23% vs 10%; *p* = 0.2278). However, only one sample was investigated in this study and a larger number of samples examined in this way in future studies will give a clearer picture.

Residue D151, which serves as a proton donor in catalytic activity and is associated with the active site of NA, is expected to affect the enzymatic reaction of NAIs [32,33]. Substitution of D151 to G or N was shown to decrease virus susceptibility to the NAIs, i.e. oseltamivir, zanamivir, and peramivir [8-10,34]. Nonetheless, the D151G and D151N mutations conferred different degrees of significantly enhanced resistance to the three NAIs, when combined with H274Y (N2 numbering) or H275Y (N1 numbering) mutation [8,9]. Thus, these mutations induced during viral culture can give a false impression of drug resistance during influenza virus surveillance. 

Furthermore, substitution of D151 to G or N can change the specificity of NA to gain receptor-binding capacity [7,35-37], besides reducing its enzymatic ability to remove sialic acids from both the SAα2,3 and SAα2,6 Gal-linked receptors of HA, to enable release of progeny from infected cells [37]. This additional acquired receptor-binding capacity can cause biased results in the hemagglutinin inhibition assay [7,38], which is frequently used by the WHO for antigenic monitoring for regular vaccine update. 

 The finding of mixed populations in most of the MDCK-isolates in our study (17/18, Table 1) is not totally unexpected and may just indicate a gradual adaptation to a new host cell species – further passages in the same cell-line would eventually yield a pure population adapted to that cell-line.

In this study, the D151 mutations were found only in MDCK-cultured isolates and not in the clinical samples. This observation could be explained by two hypotheses. The clinical samples may contain D151 mutant viruses at concentrations too low to be detected by the Sanger method. In the MDCK culture, these mutant viruses may be selectively amplified and detected. This is supported by a previous study that found up to 13% of D151 mutant population in a given clinical sample using a pyrosequencing method [7], a concentration near the detection limit of the Sanger method. On the other hand, it is also possible that the D151 mutant was induced *de novo* in the MDCK culture medium [8,11,12,39,40]. This latter hypothesis was supported by the finding of only 2/736 (0.3%) of D151 mutant virus in the clinical sample (Table 2).

Most of the laboratories that contributed influenza genetic sequences to the EpiFlu database used MDCK cell-lines for influenza virus propagation (Table 2). Interestingly, despite the differences in the number of passage history and potential methodology differences, the proportion of D151 mutations found in this study were similar to those deposited in the EpiFlu database (Table 2).

### MDCK-induced changes in the other viral proteins

Strikingly, no mutation was detected in the PA and MP gene of all the samples tested. A single sporadic synonymous mutation (N312) in the PB1 gene was observed in the 20 culture replicates of the influenza A/Singapore/H2011.704/2011(H3N2) primary clinical sample, but none were found in PB1 in the other individual, single culture pairwise comparisons. Relatively few or no MDCK-induced mutations were found in the MP, PA, and PB1 genes, suggesting that these genes are not subject to MDCK-specific adaptation selection pressures. 

 In a previous study, MDCK-induced mutations were reported in the HA and MP genes with passage histories of 3 and 10, respectively [16]. The mutations found included R83K, H183L, H156N, R220G, V226I, R229G, R229I, and R229K in the HA_1_ protein; A147T, T147A, and G150E in the HA_2_ protein; A30T in the ion channel matrix (M2) protein, but none of these mutations were detected in this study. However, it should be noted that our study employed a Sanger sequencing-based approach that could only detect the mixed viral population of as low as 10%, which could have missed the minorities that existed in less than 10% proportions in the tested samples.

Primary clinical specimens collected directly from patients vary in amount of viruses, depending on when the samples were taken post-symptom onset [41]. Even so, our study showed that the rate of MDCK-induced mutation did not correlate with the viral titers (Ct values) of the clinical samples, suggesting that this was more of a sporadic event, rather than one that was necessarily viral load dependent. This implies that the MDCK-adapted mutations were not viral load (and therefore viral replication) dependent.

In conclusion, apart from providing information on MDCK-induced mutations, this study has provided further support for the use of D151 as a marker of MDCK-cultured isolates. D151 can confound the interpretation of hemagglutinin inhibition and drug resistance results when the isolate is obtained from MDCK cultures, e.g. the WHO vaccine and drug-sensitivity surveillance programs [10,38]. This may be overcome by the exclusion of isolates with D151 mutations after genotyping. 

## Materials and Methods

### Ethics statement

All research activities involving the use of these clinical samples were reviewed and approved by the local institutional ethics review board (National Healthcare Group: B/09/360 and E/09/341). Waiver of consent was granted to this study under the following conditions: (1) only leftover clinical samples, initially submitted for diagnostic influenza testing, were used, (2) no additional clinical sample was sought from the patients for the purpose of this study (3), the clinical samples were fully anonymized prior to the sequencing analysis, and the anonymization was maintained during data analysis and publication (4) the results of this study do not impact the clinical management of these patients and are not shared with their attending physicians

### Clinical samples

Clinical samples collected in the form of nasopharyngeal, nasal or throat swabs between May 2009 and October 2011 were processed and tested using a combination of clinically validated reverse-transcription-polymerase chain reaction (RT-PCR) assays [42,43]. The Ct values of the clinical samples were determined using the in-house influenza A/B RT-PCR assay that targets the MP gene [43]. These included samples collected from patients who attended NUH or its affiliated primary care clinics. In addition, the antiviral treatment histories for patients with the D151 mutation were examined and recorded. 

### MDCK culture

Influenza A/H3N2 clinical samples with Ct values less than 33.22 were included in this study and cultured using the MDCK.2 (ATCC; CRL-2936) cell-line with a single passage history. Trypsin-treated MDCK viral culturing was performed with reference to CLSI Viral Culture; Approved Guideline [44]. Briefly, the shell vial monolayer of the culture was first rinsed with 0.5 mL minimum essential media with 0.8% trypsin (TMM) to remove traces of growth media. The clinical samples were aspirated into a 5-mL sterile syringe and filtered through a Minisart syringe filter of 0.45 μm pore size (Sartorius, Goettingen, DE) directly into the MDCK monolayer shell vials, followed by centrifugation of 760 relative centrifugal force for 30 minutes at 28 °C. The filtrates were decanted. The cell vial cultures were maintained with 1 mL of TMM and incubated at 33 °C in 5% CO_2_ for up to 7 days, until the characteristic influenza virus cytopathic effect was observed. For the 20 culture replicates, clinical sample influenza A/Singapore/H2011.704/2011(H3N2), which contained a high viral load, was diluted 50 with universal transport medium (Copan Diagnostics Inc., Corona, CA) prior to culture.

### Viral RNA

Viral RNA was extracted from 200 μL of clinical and cultured samples with either the Qiagen EZ1 Virus mini kit v2.0 or the QIAsymphony Virus/Bacteria mini kit, using their respective proprietary Bio Robot EZ1 and QIAsymphony automated platforms (Qiagen, Valencia, CA), according to the manufacturer’s instructions. All extracted RNAs were eluted into a final volume of 60 μL of elution buffer. 

### Whole genome sequencing

Genome sequencing for all samples was performed using a Sanger sequencing method that we previously described [19]. The primers used for genome-wide amplification and sequencing experiments are provided in this article [19]. In brief, genome-wide amplification was completed using 19 RT-PCRs and 39 sequencing reactions were carried out for genome-wide sequencing, as published. All sequences were assembled and verified using the ATF software, version 1.0.2.41 (Connexio Genomics, Perth, WA, Australia), using the reference sequence influenza A/Nanjing/1/2009(H3N2) for all segments (GenBank accession: GU907114- GU907117 and GU907119-GU907121), except for the PB1 segment, for which the reference sequence was influenza A/Sendai-H/F193/2007(H3N2) (GenBank accession: AB441948). The multiple A’s observed at the 3’end of the NA, NP, and PA genes were checked carefully by visualization of the sequencing chromatogram. All genome sequences generated in this study have been subjected to the mosaic detection procedure described by Lam et al. 2013 [18]. 

### Sequence comparison

Differences in nucleotide sequences between clinical and MDCK-cultured samples were identified by pairwise comparison with a program written using Statistical Analysis System 9, (SAS Institute Inc., Cary, NC, USA). Sequences of the 20 culture replicates of influenza strain A/Singapore/H2011.704/2011(H3N2) were aligned using BioEdit (version 7.1.3.0). The nucleotide and aa differences among the 20 replicates were identified by visual inspection.

### Statistical analysis

The association of Ct values in the mutation-affected and non-affected groups was examined with the 2 Sample T-test. All the categorical variables were compared using Fisher’s exact test. All the statistical analyses were performed using IBM SPSS Statistics software version 19. *P* values of <0.05 were considered statistically significant.

### GISAID EpiFlu database investigation for the D151 mutation in NA

Sample source types of all the human influenza A/H3N2 viral genome sequences that were available on the GISAID EpiFlu database and collected from Jan 2004 to Dec 2012 (as of 15 August 2013) were examined individually. All missense mutations occurring at residue 151 of the NA gene were recorded with respect to these sample source types for comparison to our study results.
